# The Association of Maternal Weight Status throughout the Life-Course with the Development of Childhood Obesity: A Secondary Analysis of the Healthy Growth Study Data

**DOI:** 10.3390/nu15214602

**Published:** 2023-10-29

**Authors:** Adriana Mannino, Katerina Sarapis, Niki Mourouti, Eva Karaglani, Costas A. Anastasiou, Yannis Manios, George Moschonis

**Affiliations:** 1 Department of Food, Nutrition and Dietetics, School Allied Health, Human Services & Sport, La Trobe University, Melbourne, VIC 3086, Australia; a.mannino@latrobe.edu.au (A.M.); k.sarapis@latrobe.edu.au (K.S.); 2 Department of Nutrition and Dietetics, School of Health Science and Education, Harokopio University, 17671 Athens, Greece; nmourouti@hua.gr (N.M.); ekaragl@hua.gr (E.K.); acostas@hua.gr (C.A.A.); manios@hua.gr (Y.M.); 3 Department of Nutrition and Dietetics, Hellenic Mediterranean University, 72300 Sitia, Greece; 4 Institute of Agri-Food and Life Sciences, Hellenic Mediterranean University Research Centre, 71410 Heraklion, Greece

**Keywords:** childhood obesity, maternal overweight, maternal obesity, pre-pregnancy, gestational weight gain, perinatal factors

## Abstract

Maternal weight-status at various time-points may influence child obesity development, however the most critical time-point remains unidentified. We used data from the Healthy Growth Study, a cross-sectional study of 2666 Greek schoolchildren aged 9–13 years, exploring associations between childhood obesity and maternal weight-status at pre-pregnancy, during pregnancy/gestational weight gain, and at the child’s pre-adolescence. Logistic regression analyses examined associations between maternal weight-status being “below” or “above” the recommended cut-off points (WHO BMI thresholds or IOM cut-off points), at the three time-points, individually or combined into weight-status trajectory groups to determine the strongest associations with child obesity in pre-adolescence. Adjusted models found significant associations and the highest odds ratios [95% Confidence Intervals] for mothers affected by obesity before pregnancy (4.16 [2.47, 7.02]), those with excessive gestational weight gain during pregnancy (1.50 [1.08, 2.08]), and those affected by obesity at their child’s pre-adolescence (3.3 [2.29, 4.87]). When combining these weight-status groups, mothers who were above–above–below (3.24 [1.10, 9.55]), and above–above–above (3.07 [1.95, 4.85]) the healthy weight recommendation-based thresholds in each time-point, had a three-fold higher likelihood of child obesity, compared to the below–below–below trajectory group. Maternal obesity across all examined time-points was significantly associated with childhood obesity. Effective childhood obesity preventive initiatives should commence at pre-conception, targeting maternal weight throughout the life-course and childhood developmental stages.

## 1. Introduction

Obesity is a complicated, prevalent, and multifactorial chronic condition [1]. It has grown to be one of the most critical public health problems worldwide, with 39% of the global adult population being overweight, and 13% obese [2]. In alignment with the global trend, the prevalence of overweight and obesity in Europe is also considerably high, (36% overweight and 17% obese) [3], mainly in the southern European countries [4,5]. In Greece alone, 35% of adults have been reported to be overweight and 27% obese [5,6]. Obesity is also very prevalent in younger ages; according to 2016 data, this clinical condition affects over 124 million children and adolescents (5–19 years of age) globally [2]. In 2019, it was estimated that over 40% of school children in Greece were overweight and obese [4]. Moreover, according to the World Health Organization (WHO) data, in 2016, the rate of obesity alone for children and adolescents in Greece was 13.8% [7].

There are multiple non-modifiable and modifiable risk factors contributing to the development of obesity, with some of these factors falling into both categories. The main non-modifiable risk factors include age, gender, puberty, developmental conditions, heritable factors (genetics and epigenetics), ethnicity, and other factors which may also be considered to be potentially modifiable, i.e., income, education, employment status and neighbourhood composition [1,8,9,10]. Conversely, a wide range of modifiable risk factors such as psychosocial, behavioural, and environmental factors have been associated with obesity, thus contributing to an “obesogenic environment” [11,12]. The latter is considered as a key influence of the modifiable behavioural risk factors that are likely to increase the risk of childhood obesity [1,13]. For children and adolescents, the age of obesity onset is also an important factor influencing long-term health. Obesity at the age of pre-adolescence (9–12 years) increases the risk to develop type 2 diabetes, cardio-metabolic diseases, and mortality [14,15,16].

Studies have also shown that children are at greater risk of overweight or obesity when parents also have an unhealthy BMI [17,18,19] since they are more likely to create and sustain a permanent positive energy balance within the home [1,13]. In this regard, children adopt obesogenic behaviours with early life exposure to sedentary habits, low physical activity levels, increased screen time, and low-quality dietary patterns (i.e., high consumption of energy-dense processed foods and low consumption of vegetables and wholemeal foods) [17,18,19]. Mothers have shown to play a key role of influence, since they are pivotal in setting the food landscape and eating patterns/choices of their children [19,20]. There is also considerable evidence pointing to perinatal factors occurring prior to the child’s conception and risk of obesity [21,22,23], as well as during pregnancy [24,25,26]. Throughout the time-points of before pregnancy and during pregnancy up to the child’s birth, there are several mechanistic pathways occurring early in life that have been proposed to interpret the associations observed between maternal obesity with the occurrence of obesity in offspring. Perinatal factors occurring before pregnancy and in the intrauterine environment have been found to result in physiological and metabolic changes, impacting the long-term health of children [27,28,29].

Recently our research team conducted a systematic literature review [30], which demonstrated that the current evidence does not conclude which maternal exposure (pre-pregnancy or during childhood) has a stronger effect on the development of childhood obesity. Although an extensive volume of literature suggests maternal weight status at individual time-points as a risk factor for childhood obesity or combinations of time-points, for example pre-pregnancy [23,29,31,32,33,34], during pregnancy [35,36], during infancy [37], and during childhood [38,39], only a handful of studies have reported the combined effect of these exposures at different time-points [32,40,41]. There is scarce evidence combining each of these exposure time-points with maternal obesity during the child’s life, i.e., pre-pregnancy, during pregnancy/gestational weight gain, and maternal weight status at their offspring’s pre-adolescence. Furthermore, to our knowledge there have been no studies that have combined pre-pregnancy, gestational weight gain, and maternal weight status at pre-adolescence and examined all possible combinations of these exposures on the likelihood of childhood obesity.

To address the current knowledge gap and to further understand the most appropriate timing of preventative strategies, this study aimed to test associations between childhood obesity and maternal weight status at three different time-points in the life course of mothers, i.e., before pregnancy, during pregnancy (i.e., gestational weight gain), and at their child’s pre-adolescence.

## 2. Materials and Methods

### 2.1. Study Design and Population

This secondary analysis used data from the Healthy Growth Study, a nationally representative cross sectional study, with retrospectively collected data on perinatal risk factors, initiated by way of a pilot study in May 2007 and concluded in 2009 [27,42]. The Healthy Growth Study comprised school children attending the 5th and 6th grade (aged 9–13 years, *n* = 2666; boys *n* = 1348; girls *n* = 1318 [with signed consent]). School children were recruited from municipalities within 4 counties across the wider urban area of Athens, Greece; counties included Thessaloniki, Attica, Aitoloakarnania, and Heraklion. Schools were identified and invited to participate in the study (*n* = 77) as per data obtained from the Greek Ministry of Education. All schools responded positively [27]. A detailed process of sampling for the Healthy Growth Study can be found elsewhere [27,42]. The Healthy Growth Study adhered to the Declaration of Helsinki and the conventions of the Council of Europe on Human Rights and Biomedicine. The study was approved by the Greek Ministry of National Education and the Ethics Committee of Harokopio University of Athens. The present secondary data analysis of the Healthy Growth Study was approved by La Trobe University Human Research Ethics Committee on 29 July 2022 (Ethics Approval Number: HEC22199).

### 2.2. Family and Sociodemographic Data

Socio-economic and demographic background information of participants and their families was collected during face-to-face interviews at schools with guardians by rigorously trained interviewers with the use of a standardised questionnaire to reduce interviewer bias. Demographic data were collected from parents and guardians, including age of parents, years of education, nationality, and region of residence (i.e., urban vs. rural). Social-related data were also collected, including the primary guardian(s) of the child. Finally, economic characteristics were collected, which included employment status [27].

### 2.3. Physical Activity Levels of Children

To calculate the average of total steps per day, participants wore waist-mounted pedometers (vertically aligned with the patella) (Yamax SW-200 Digiwalker, Tokyo, Japan) for 7 days. A standardised interview was used to assess physical activity during leisure time, on two weekdays and one weekend, based on a valid questionnaire [21,43]. Physical activities (both leisure and organised) were grouped into moderate-to-vigorous physical activities (MVPA) (intensity higher than four metabolic equivalents, METs). Organised moderate-to-vigorous physical activities (OMVPA) was a fraction of total MVPA and were defined as activities that took place in an organised manner i.e., under the supervision of a trainer or coach. Screen time was also assessed via reports from children regarding their television/video viewing time and time playing computer/video games and reported by calculating the average screentime over a week. Detailed description of the physical activity data collection methods is reported elsewhere [21,43].

### 2.4. Perinatal Data

Perinatal data were collected from guardians who recalled and reported the information on their child’s growth during infancy, such as the growth rate during the first 6 months of life. Growth velocity was defined by calculating changes in weight or height of infants over time and was categorised as normal growth, poor growth, and rapid growth as per the International Obesity Task Force (IOTF) cut-offs [44]. Birth weight and gestational age were extracted from each child’s birth certificate and medical record, and were used to calculate birth weight for age z-score, which allowed the classification of children at birth as small for gestational age (SGA, <10th percentile), appropriate for gestational age (AGA, 10 to 89th percentile), and large for gestational age (LGA, ≥90th percentile) [22,27].

Pre-pregnancy data (i.e., parity and history of miscarriages before child’s birth); gestational data (i.e., history of smoking, alcohol use, gestational diabetes mellitus and high blood pressure during pregnancy, type of delivery (vaginal vs. caesarean); and factors occurring at their child’s pre-adolescence, including infant’s feeding practices from birth to 6 months of age, i.e., exclusive breastfeeding vs. use of formula or mixed feeding; history of maternal smoking or alcohol use during breastfeeding; age of the initiation of solids in infants’ diet; and parental smoking at their child’s pre-adolescence were also collected [22,27]. Finally, anthropometric data of parents, which included body weight and height, were also reported by mothers and fathers.

Mothers’ anthropometrics before pregnancy and at their child’s pre-adolescence were used to calculate their body mass index (BMI), which was used to categorise them as ‘underweight’ (<18.5 kg/m^2^, ‘healthy weight’ (18.50–24.99 kg/m^2^), ‘overweight’ (25.00–29.99 kg/m^2^), or ‘obese’ (≥30.00 kg/m^2^), based on WHO’s weight status classification of adults [45]. Mothers’ gestational weight gain was categorised based on the Institute of Medicine (IOM) classifications of ‘weight gain in mothers with a pre-pregnancy BMI category of underweight (12.5–18 kg), healthy-weight (11.5–16 kg), overweight (7–11.5 kg), and obese (5–9 kg)’ [46].

### 2.5. Maternal Recommendation-Based Weight Status Trajectory Groups

To create ‘maternal recommendation-based weight status trajectory groups’, we followed the procedure set out by Leonard, Rasmussen, King, and Abrams [41]. According to this procedure, we dichotomised each mother’s self-reported pre-pregnancy BMI (overweight or obese: ≥25 kg/m^2^ [45]), gestational weight gain from clinical records (excessive based on the IOM guidelines: ≥18 kg if underweight, ≥16 kg if healthy weight, ≥11.5 kg if overweight, or ≥9 kg if obese), and self-reported current maternal BMI (maternal BMI at the child’s current age [pre-adolescence] (overweight or obese: ≥25 kg/m^2^) [46].

For each timepoint, in the following order, pre-pregnancy, gestational weight gain, and at the child’s pre-adolescence, mothers were then categorised as “above” or “below” the WHO (2000) BMI thresholds or IOM (2009) cut-off points, and these categories were combined into all possible combinations of the eight maternal recommendation-based weight status trajectory groups, assigned as follows: below–below–below (reference group), below–below–above, below–above–below, below–above–above, above–below–below, above–below–above, above–above–below, and above–above–above. For the above category, this has been described in the results as ‘above’ or ‘upper’ threshold. “Above” includes maternal weight status above the WHO classification of adults according to BMI thresholds (overweight/obesity) [45] or IOM recommendations [46]). Below includes maternal weight status as healthy or below the WHO classification of adults [45] according to BMI, or IOM recommendations [46].

### 2.6. Anthropometric Measurements of Children

For school children with parental signed informed consent, a full medical examination was conducted, which included anthropometric and body composition measurements, blood collection and clinical examination. The physical examination was carried out by two trained members of the research team with identical protocols and equipment used across all schools [27]. Regarding body weight, children were weighed without shoes and in the minimum clothing possible, to the nearest 10 g using a Seca digital scale (Seca Alpha, Model 770, Hamburg, Germany).

Height was measured without shoes, with shoulders relaxed, arms hanging freely, and participants heads aligned in Frankfort plane, to the nearest 0.1 cm using a commercial stadiometer (Leicester Height Measure, Invicta Plastics Ltd., Oadby, UK). The measures of children’s weight and height were converted to BMI using Quetelet’s equation (weight (kg)/height^2^ (m^2^)) [47]. Waist circumference (WC) was measured, with the child standing, to the nearest 0.1 cm at the end of a gentle expiration, with the use of a Hoechstmass non-elastic tape. The measuring tape was placed around the waist, half-way between the lower rib margin and the iliac crest. Hip circumference was measured to the nearest 0.1 cm, at the level of greater trochanters and pubic symphysis. Waist-to-Hip ratio (WHR) (waist circumference/hip circumference) was also calculated for each school child [27].

The IOTF BMI-for-age cut-off points were used to categorise school children as “underweight”, “ healthy weight”, “overweight”, or “obese” [44]. Waist circumference measurements were classified based on age and sex-specific percentiles (≥90th percentile) to identify central obesity [48,49,50].

### 2.7. Statistical Analyses

All analyses were based on the Healthy Growth Study population, which included selected cases, comprising the majority of the total sample, but excluding those with energy intakes of <800 kilocalories (kcal)/day and >3000 kcal/day, as a strategy to exclude under- and over-reporters of dietary intake [51]. Both continuous and categorical variables were used in the current analysis. Logistic regression analysis was used to examine adjusted associations between maternal weight status at each one of the three time-points (i.e., pre-pregnancy, during pregnancy, and at their child’s pre-adolescence) separately or combined into recommendation-based weight status trajectory groups to test all possible combinations of these groups, and the outcome of child obesity. The level of statistical significance was set at *p* < 0.05 and the reported *p*-values were two-tailed. All statistical analyses were performed using the IBM SPSS Statistics package, version 28.0, for Windows (IMB, Armonk, NY, USA). Figures were created with the use of Graphpad (Prism), version 9 for Windows (Dotmatics, San Diego, CA, USA).

### 2.8. Confounding Variables

According to the available evidence pertaining to factors which might affect the examined associations, specific variables collected for different time-points in the life-course of children and their mothers were included as possible confounders in the regression models [27,29,52]. More specifically, confounding variables related to children’s early life included the three categories of birth weight (i.e., SGA, appropriate for gestational age (AGA) and LGA) and growth velocity from birth to the first 6 months of life (i.e., normal growth, poor growth, and rapid growth); infant feeding history (i.e., exclusive breastfeeding, exclusive formula, mixed feeing); infant’s age of solid foods introduction, in months; and gestational age (in months). Regarding variables that can also serve as confounders and were collected from children in their pre-adolescence, these included their average dietary energy intake (in Kcal/day) and their daily moderate-to-vigorous physical activity (in minutes/day). Regarding confounding variables related to parents, these included mother’s and father’s age (in years), three categories of mother’s and father’s education (i.e., <9 years, 9–12 years, >12 years), mother’s age at birth (in years), and father’s BMI (in kg/m^2^).

## 3. Results

### 3.1. Socio-Demographic and Anthropometric Characteristics

Table 1 presents the socio-demographic characteristics of the study participants in the Healthy Growth Study. Of the 2519 children included, 50.4% were girls. The mean age of children was 11.2 ± 0.67 years.

Table 2 presents the anthropometric characteristics of study participants. Girls were taller (*p* = 0.010) and had a wider hip circumference (*p* = 0.022) than boys. Boys had higher BMIs and waist-to-hip ratios than girls, i.e., 20.5 ± 3.89 vs. 20.1 ± 3.69 for BMI (*p* = 0.010), and 87.0 ± 5.71 vs. 85.4 ± 7.28 (*p* < 0.001) for waist-to-hip ratios, respectively. No other statistically significant gender differences were observed.

### 3.2. Perinatal Characteristics

Perinatal characteristics of study participants are presented in Table 3. Most children (81.1%) had a gestational age (at birth) of 37 weeks or over, with 80.7% born with an appropriate gestational weight (i.e., AGA), while 7.4% were LGA. With regards to growth velocity, 32.8% of children had a rapid growth and 56% of children had a normal growth. With regards to factors relating to the mother, 15% of mothers had miscarried prior to the child’s pregnancy, and most mothers did not drink alcohol (98.1%), smoke (84.1%), have high blood pressure (94.6%), or have gestational diabetes (95.1%) during pregnancy.

For factors related to children after birth, most children were born by vaginal delivery (71.6%), most children were mixed fed via a combination of breastfeeding and formula (72.7%) and commenced solid foods at 5–6 months of age (66.6%). Regarding mothers, 99.0% reported not drinking alcohol or smoking during breastfeeding, however 39.2% of mothers smoked as the child grew up, and 45.1% of parents smoked at their child’s pre-adolescence. With regards to gender differences, girls had a higher rapid growth velocity than boys 36.3% vs. 29.1%, *p* = 0.002. Additionally, girls were more likely to have mothers who reported they did not know of their gestational diabetes history during pregnancy, 3.2% vs. 2.8%, *p* = 0.033.

### 3.3. Weight Status Prevalence

**Figure 1:**
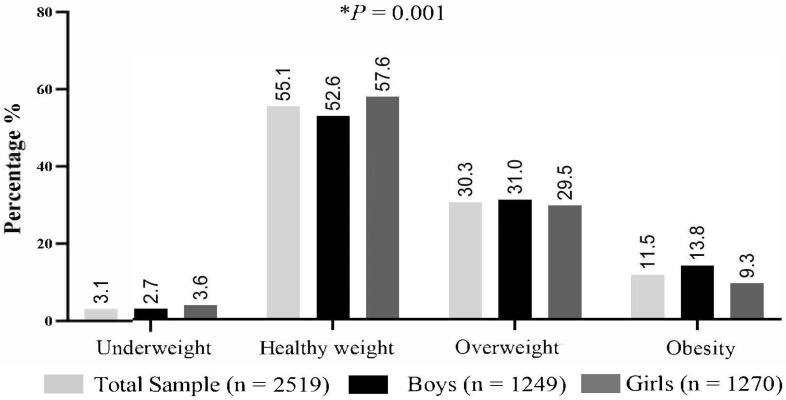
Child weight status in sample population. Abbreviations: %, percentage; *n*, sample size. Child weight status as defined by the international body mass index cut-offs for thinness, overweight and obesity (IOTF) [44,53]. The asterisk (*) indicates statistically significant difference between boys and girls.

The prevalence of underweight, healthy weight, overweight, and obesity among participants in the study is presented in Figure 1. The prevalence of overweight and obesity in the total sample of school children was 30.3% and 11.5%, respectively. The prevalence rates of overweight and obesity were higher in boys compared to girls, with 31.0% vs. 29.5% (although not statistically significant) for overweight, and 13.8% vs. 9.3% (*p* <0.05) for obesity, respectively. The prevalence of underweight, healthy weight, overweight, and obesity among mothers at the various time-points categories are available in Supplementary Figure S1.

### 3.4. Associations between Maternal Weight Status and Child Obesity

The logistic regressions models that assessed the associations between maternal weight status and child obesity status were stratified by gender, while the analyses were also adjusted for a number of potential confounders. The results from the logistic regressions models are presented as odds ratios and 95% confidence intervals in Table 4.

According to the observed associations, maternal pre-pregnancy overweight was associated with an increased likelihood of child obesity, by 1.90 (1.34, 2.70) in the total sample, 1.65 (1.00, 2.72) in boys, and 2.43 (1.45, 4.08) in girls. However significant and even stronger associations were also found with maternal pre-pregnancy obesity, which increased the likelihood of child obesity by 4.16 (2.47, 7.02) in the total sample, 3.55 (1.68, 7.02) in boys, and 5.60 (2.60, 12.08) in girls.

For gestational weight gain, maternal weight gain above the IOM recommendations, was associated with an increased likelihood of childhood obesity by 1.50 (1.08, 2.08) in the total sample and 1.66 (1.07, 3.56) in boys, while no significant associations were observed in girls. Similarly, maternal obesity at their child’s pre-adolescence was associated with an increased likelihood of child obesity by 3.34 (2.29, 4.87) in the total sample, 3.14 (1.89, 5.42) in boys, and 4.18 (2.31, 7.56) in girls.

The highest odds ratios in these associations with child obesity were observed for mothers who were affected by overweight or obesity in each time-point, and more specifically for mothers affected by obesity prior to conception, followed by mothers who were affected by obesity at their child’s pre-adolescence.

### 3.5. Associations between Recommendation-Based Maternal Weight Trajectory Groups and Child Obesity

**Figure 2:**
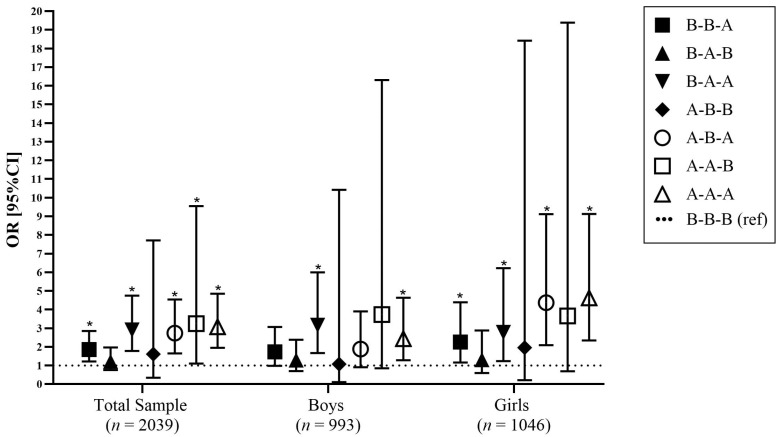
Adjusted associations of maternal weight status trajectories and childhood obesity. Markers indicate odds ratios and lines indicate 95% CIs, adjusted for confounders. * *p* < 0.05. Abbreviations: B, below (includes maternal weight status as healthy or below the WHO classification of adults according to BMI [45], or Institute of Medicine (IOM) recommendations [46]); A, above (includes maternal weight status above the WHO classification of adults according to BMI thresholds (overweight/obese) [45] or IOM recommendations [46]). Maternal weight trajectories groups have been combined based on recommended cut-off points for weight status at the following time-points pre-pregnancy and during pregnancy (gestational weight gain [based on the IOM (2009) recommendations [46]]), and, at their child’s pre-adolescence, combined into 8 maternal weight trajectory groups; OR, Odds Ratio; 95%CI, 95% Confidence Interval.

The odds ratios and 95% CIs that reflect the associations between recommendation-based maternal weight status trajectories and child obesity status are presented in Figure 2, after adjusting for a number of potential confounders. According to these findings, there were statistically significant positive associations observed for mothers with weight status above the WHO (2000) BMI thresholds or IOM (2009) cut-off points, healthy thresholds in each of the examined time-points, thus showing an increased likelihood for child obesity, i.e., by 3.1 times in the total sample (1.95, 4.85). 2.4 times in boys (1.28, 4.63), and 4.6 times in girls (2.34, 9.12).

Overall, girls were found to have the highest odds ratio in the above–above–above category (4.62 [2.34, 9.12]). There were specific time-points that, when combined, could also create a higher risk for childhood obesity, compared to children with mothers within the healthy ranges or reference ranges. Specifically, there were higher odds ratios for children born to mothers who were above thresholds for at least one of the maternal time-points investigated. More specifically, for mothers who were above the healthy threshold at their child’s pre-adolescence, the likelihood for childhood obesity increased by 1.9 times in the total sample (1.21, 2.84), 1.7 times in boys (0.98, 3.06), and 2.3 times in girls (1.16, 4.39).

Furthermore, when maternal weight status was above during pregnancy and at their child’s pre-adolescence, the likelihood of childhood obesity increased by 2.9 times in the total sample (1.78, 4.74), 3.2 times in boys (1.67, 5.99), and 2.8 times in girls (1.23, 6.21). For mothers above the healthy threshold before pregnancy and at their child’s pre-adolescence, the likelihood of childhood obesity increased by 2.7 times in the total sample (1.65, 4.54), 1.8 times in boys (0.91, 3.90) and 4.4 times in girls (2.09, 9.12).

## 4. Discussion

To our knowledge this is the first study to test associations between pre-pregnancy maternal overweight/obesity, excessive gestational weight gain, and maternal overweight/obesity at their child’s pre-adolescence with child obesity. More specifically, the present study reported strong associations and the highest odds ratios for mothers who were above (the WHO (2000) BMI thresholds or IOM (2009) cut-off points) recommendation-based weight status thresholds in each time-point and child obesity. In addition, when these time-points were combined, the highest rates of child obesity were found among women who were above the recommendation-based thresholds in two or all three of the time-points combined, where there was a three-fold increase in the likelihood of child obesity in the total sample. The findings from this study suggest that maternal overweight and obesity across all time-points examined in this study are important risk factors for the development of child and adolescent obesity.

When we compared maternal weight status in association with childhood obesity, we found the highest odds ratios for children, with mothers who were affected by obesity before pregnancy (a four-fold likelihood). This finding is consistent with the results of previous studies, including big data analytics, exploring the association between maternal pre-pregnancy obesity and childhood obesity [29,54,55,56,57]. The study by Leonard, Rasmussen, King, and Abrams [41] examined similar maternal exposures, i.e., pre-pregnancy, gestational weight gain and postpartum weight retention and their associations with child obesity. They found pre-pregnancy overweight/obesity to be associated with more than a two-fold increase in the risk in childhood obesity (aged 6–19 years) [41]. The presence of overweight and obesity prior to pregnancy can be influenced by a number of factors, including nutritional status, increase of fat accumulation, inflammation, and maternal genetic pre-disposition [55,58,59]. Obesity is an inflammatory state associated with insulin resistance and increasing blood glucose levels. After conception, this exposes the growing foetus to an over-nutrition environment in utero [55,58], resulting in a cascade of changes to foetal developmental programming and subsequently increasing the risk of obesity in offspring [29,56].

Excessive gestational weight gain is considered one of the risk factors for child obesity [35,40,60,61,62]. The present study found that children and adolescents were 1.5 times more likely to be affected by obesity if their mothers gained weight above the IOM recommendations during pregnancy. Similar findings were highlighted by Baran et al. [63] who found that mothers’ gestational weight gain was higher in children affected by obesity at ages 7–11, compared to children of healthy weight status. Our findings also support the literature that has previously compared the effect of maternal pre-pregnancy overweight or obesity and excessive gestational weight gain, showing that pre-pregnancy weight status is a stronger predictor for child obesity [29,57]. According to models and hypotheses that attempt to interpret the early origins of obesity and chronic disease, both the presence of maternal pre-pregnancy overweight/obesity and excessive gestational weight gain shape an intrauterine risk environment for the growing foetus, influencing physiological and metabolic adaptions, which can negatively affect the long term health of offspring [64]. These adaptions occur in response to a derivative environment, and their primary aim is to increase the survival of the growing foetus [59]. As postulated by the Thrifty Phenotype hypothesis, these adaptions, or changes to the in-utero programming, increases the foetus capacity to store calories, and therefore increase the likelihood of overweight and obesity in later life and as part of an environment with adequate food availability [59].

The presence of maternal overweight and obesity during childhood has been reported as a risk factor for child obesity [39,65,66,67]. In this context, the present study also showed that maternal overweight/obesity at their child’s pre-adolescence was associated with high odds of child and adolescent obesity, second to those found for the pre-pregnancy. The retrospective cohort study by Vehapoglu, Goknar, Turel, Torun, and Ozgurhan [67] found a 4-fold increase in the likelihood of child obesity when mothers were also affected by obesity. Similarly, a prospective cohort study by Kjaer, Faurholt-Jepsen, Medrano, Elwan, Mehta, Christensen, and Wojcicki [66] reported that obesity in mothers 5 years post-partum was associated with child obesity at 9 years. Mothers are more likely to be the primary caregiver of their child, thus having a key role in influencing the energy-balance-related behaviours of their children [39]; this includes influence over food type and choice, the promotion of physical activity, sleep patterns, screen time, and other lifestyle choices [12,21,27,48]. The factors contributing to the increased weight status of mothers would also have greater influence over the child’s weight status, hence increasing the likelihood of child obesity [37,68]. With mothers affected by overweight and obesity likely creating and sustaining the obesogenic environment, this reinforces the Maternal Resource Hypothesis [69] and further delineates this as a strong risk factor for child and adolescent obesity [70]. This further highlights the importance of maternal weight status on their children’s weight status and the risk of childhood obesity and related comorbidities [71].

Most of the current literature has examined the effect of maternal overweight and obesity on child obesity at independent time-points, however there is scarce evidence combinedly examining maternal weight status in different time-points. The present study showed a cumulative positive association when combining maternal overweight and excess weight gain in critical time-points with the likelihood of child obesity, observing the highest odds ratios when there were any two and especially three time-points with maternal weight status above the recommendation-based weight status thresholds combined. The multi-etiological/dual-determination model of child obesity could explain these findings, where genetic and perinatal influences meet the obesogenic environment, all influencing cues of overeating [9,72].

This study further highlights the importance of maternal weight status before, during and after pregnancy on the risk of childhood obesity and places special emphasis on the transitioning of children to adolescence (i.e., on the life stage of pre-adolescence). For children and adolescents, the age of obesity onset is an important factor influencing long-term health [14,15,16]. More specifically, there are important hormonal (i.e., oestradiol and gonadotropin-releasing hormone) [73], and biomarker changes (i.e., serum lipids, glucose, and insulin levels), particularly in girls [74,75,76], occurring in this transitional stage, which may have a stronger influence on metabolism compared to current eating behaviours. With overweight and obesity during childhood, if an individual’s BMI further increases during the pre-adolescence stage, the risk of poor health outcomes is strengthened, and it becomes a further risk if this increase continues into the adolescence [16,77,78]. There are also other environmental changes occurring during this transitional stage, where the sphere of the obesogenic environment expands to social influences [79]. Obesity as well as overweight have long-lasting adverse physical, mental, and emotional effects on children as they move into adolescence and then to adulthood [13]. Previous intervention studies have demonstrated a decrease in the risk of obesity in adolescence and adulthood when there is a reduction in body weight prior to the pre-adolescence stage [16].

In order to curb the prevalence of obesity for both mothers and children, this study provides indications on particular stages throughout the maternal life-course where interventions should take place. For the time-point of pre-conception, it is recommended that future mothers are supported through an integrated approach, including planning and preparation, which should include raising awareness about the importance of a healthy weight status and the impact of weight status above the recommendations on offspring [80]. Primary health, medical practitioners and allied health professionals should aim to support women planning pregnancies to achieve an appropriate body weight prior to conception, on the importance of physical activity, and to provide nutrition education appropriate for both mothers and children [81]. During pregnancy, the immediate environment around the expecting mother grows to a larger network of family and health professionals, who should provide appropriate support to maintain/achieve healthy weight gain throughout pregnancy, i.e., within the IOM recommendations [80,81]. It is equally important to support mothers to achieve a healthy body weight post-partum and manage their weight as their child grows up [82,83]. It is also vitally important to target families as a whole, since a recent umbrella review showed that family interventions that include nutrition and physical activity education are quite successful in improving health outcomes for children [84].

Our findings should be interpreted in light of this study’s strengths and limitations. Regarding strengths, the Healthy Growth Study provides sufficient representativeness, as it was the first large-scale epidemiological study covering the central, northern, southern, and western parts of the Greek territory, and varying social–demographic groups [27]. The sample used pre-adolescent children, which is a pivotal age as it is representative of a transition life stage from childhood to adolescence [79]. To avoid the inaccurate reporting of self-reported data, the Healthy Growth Study used trained researchers and standardised procedures to measure and collect anthropometric data, thus increasing the accuracy of determined weight status among participants. In addition, the current study reported associations that were independent of the confounding effect of third factors. The final strength is the use of longitudinal trajectories for maternal weight status across the life-course which is a recently reported approach in the examination of child obesity [41]; this study is the first to combine not only pre-pregnancy maternal weight status and gestational weight gain, but also maternal weight status at the time-point of their child’s pre-adolescence, providing a comprehensive assessment of the perinatal and obesogenic factors affecting the development of child obesity.

This study also has limitations, with its cross-sectional design representing its main limitation, as it cannot support causality in the observed associations. In addition, and although these findings are also not generalisable to other countries/populations around the world, it is important to note that the prevalence of overweight and obesity in Greece is one of the highest in the world, demonstrating the importance of determining the maternal, perinatal, and environmental factors in this population. Another limitation also lies in the fact that mothers were asked to provide information retrospectively for a period of years prior to the study i.e., pre-pregnancy, which could increase the risk of recall bias and cause a misclassification of weight status and therefore maternal weight status trajectories.

## 5. Conclusions

The present study provides a clear indication that the presence of maternal obesity in different life stages and specifically before pregnancy, during pregnancy (gestational weight gain), and at the child’s pre-adolescence, as important risk factors for the development of childhood obesity. Future birth cohort studies are needed to thoroughly examine these associations and identify the most critical time-points of maternal weight status and their effect on the development of child obesity. Health practitioners and policy makers should ensure future mothers are targeted early, while continuous messaging should be provided to pregnant women and mothers of children throughout their life-course on the importance of healthy weight maintenance not only for themselves, but for their children too.

## Figures and Tables

**Table 1:** Socio-demographic descriptive characteristics of study participants in the Healthy Growth Study.

	Total Sample (*n* = 2519 ^a^)	Boys (*n* = 1249)	Girls (*n* = 1270)	*p*-Value
	Mean ± SD	Mean ± SD	Mean ± SD	
Age (years)	11.2 ± 0.67	11.2 ± 0.7	11.2 ± 0.7	0.999 *
		%	%	
Sex		49.6	50.4	
Study region	*n* (%)	*n* (%)	*n* (%)	
Attica	1354 (53.9)	690 (55.4)	664 (52.5)	0.478
Aitoloakarnania	432 (17.2)	208 (16.7)	224 (17.7)	
Heraklion	420 (16.7)	205 (16.5)	215 (17.0)	
Thessaloniki	304 (12.1)	142 (11.4)	162 (12.8)	
Urbanisation degree	*n* (%)	*n* (%)	*n* (%)	
Metropolitan	1630 (64.7)	826 (66.1)	804 (63.3)	0.323
Semi-Urban	422 (16.8)	199 (15.9)	223 (17.9)	
Rural	467 (18.5)	224 (17.9)	243 (19.1)	
Ethnicity	*n* (%)	*n* (%)	*n* (%)	
National	2136 (84.8)	**1077 (86.3)**	1059 (83.4)	**0.042**
Non-national	382 (15.2)	171 (13.7)	**211 (16.6)**	
Primary guardian type	*n* (%)	*n* (%)	*n* (%)	
Mother	2322 (92.2)	1142 (91.4)	1180 (92.9)	0.198
Other	197 (7.8)	107 (8.6)	90 (7.1)	
Maternal education	*n* (%)	*n* (%)	*n* (%)	
<9 years	530 (21.7)	239 (19.8)	**291 (23.6)**	**0.054**
9–12 years	960 (39.3)	488 (40.4)	472 (38.2)	
>12 years	954 (39.0)	482 (39.9)	472 (38.2)	
Father’s education	*n* (%)	*n* (%)	*n* (%)	
<9 years	644 (26.1)	313 (25.6)	331 (26.5)	0.914
9–12 years	948 (38.4)	472 (38.6)	476 (38.1)	
>12 years	879 (35.6)	437 (35.8)	442 (25.4)	
Mother’s employment status	*n* (%)	*n* (%)	*n* (%)	
Unemployed	759 (33.9)	382 (34.9)	377 (33.0)	0.327
Employed	1479 (66.1)	712 (65.1)	767 (67.0)	
Parent’s age	Mean ± SD	Mean ± SD	Mean ± SD	
Mothers	39.8 ± 4.9	40.00 ± 4.8	39.6 ± 4.99	0.087 *
Fathers	44.1 ± 5.4	44.2 ± 5.3	44.2 ± 5.4	0.970 *
Parent’s weight	Mean ± SD	Mean ± SD	Mean ± SD	
Mothers (Kgs)	66.8 ± 12.6	66.9 ± 12.7	66.7 ± 12.5	0.649 *
Fathers (Kgs)	85.6 ± 12.2	85.5 ± 13.3	85.8 ± 13.2	0.611 *

Abbreviations: %, percentage; ±, plus/minus; SD, Standard Deviation; Kgs, Kilograms. ^a^ Subsample with selected cases, filtered for energy intake (excluding those with energy intakes of <800 kilocalories (kcal)/day and >3000 kcal/day). *p* values for examining associations between categorical variables were derived from the Chi-square test. * *p* values (<0.05) for testing between-group differences in continuous variables were derived from the independent samples *t*-test. Results in bold indicate statistical significance (*p* < 0.05).

**Table 2:** Anthropometric characteristics of study participants in the Healthy Growth Study.

	Total Sample (*n* = 2519 ^a^)	Boys (*n* = 1249)	Girls (*n* = 1270)	*p*-Value ^b^
	Mean ± SD	Mean ± SD	Mean ± SD	
Body weight, kg	45.3 ± 11.0	45.4 ± 11.07	45.2 ± 10.9	0.621
Height, cm	148.7 ± 7.86	**148.2 ± 7.45**	**149.2 ± 8.22**	**0.001**
BMI (kg/m^2^)	20.3 ± 3.79	**20.5 ± 3.89**	**20.1 ± 3.69**	**0.010**
Waist circumference, cm	72.9 ± 10.8	73.2 ± 10.88	72.5 ± 10.6	0.136
Hip circumference, cm	84.4 ± 9.61	**83.9 ± 9.38**	**84.7 ± 9.44**	**0.022**
Waist-to-hip ratio, %	86.2 ± 6.60	**87.0 ± 5.71**	**85.4 ± 7.28**	**<0.001**
Right arm circumference, cm	23.8 ± 3.74	23.9 ± 3.90	23.7 ± 3.60	0.129
Fat mass, kg	14.25 ± 7.63	14.2 ± 7.70	14.5 ± 7.52	0.342
WAZ: Weight for age z-score at birth	−0.15 ± 1.14	−0.15 ± 1.11	−0.14 ± 1.18	0.882
Waist Circumference Cut-offs	*n* (%)	*n* (%)	*n* (%)	
Healthy	2051 (84.4)	1006 (83.5)	1045 (85.3)	0.216
Central Obesity	379 (15.6)	199 (16.5)	180 (14.7)	

Abbreviations: ±, plus/minus; SD, Standard Deviation; kg, kilo grams; m, metres; *n*, number; %, percentage; Central obesity, defined as (≥90th percentile). ^a^ Subsample with selected cases, filtered for energy intake (excluding those with energy intakes of <800 kcal/day and >3000 kcal/day). ^b^ All *p*-values were derived from independent sample *t*-test and indicate the statistical significance of the differences between boys and girls. Results in bold indicate statistical significance (*p* < 0.05).

**Table 3:** Perinatal descriptive characteristics of study participants in the Healthy Growth Study.

	Total Sample (*n* = 2519 ^a^)	Boys (*n* = 1249)	Girls (*n* = 1270)	*p*-Value ^b^
	Mean ± SD	Mean ± SD	Mean ± SD	
**Child Growth Factors**				
Gestational age at birth				
<37 weeks	442 (18.9)	197 (18.1)	225 (19.8)	0.316
≥37 Weeks	1806 (81.1)	892 (81.9)	914 (80.2)	
Birth weight for age z-score				
AGA	1799 (80.7)	888 (81.5)	911 (80.0)	0.192
SGA	265 (11.9)	132 (12.1)	133 (11.7)	
LGA	164 (7.4)	69 (6.3)	95 (8.3)	
Growth Velocity				
Normal Growth	1236 (56.6)	**638 (59.7)**	598 (53.7)	**0.002**
Poor Growth	231 (10.6)	120 (11.2)	111 (10.0)	
Rapid Growth	715 (32.8)	311 (29.1)	**404 (36.3)**	
**Pre-pregnancy Factors**				
Parity				
Uniparous	1351 (54.5)	687 (56.0)	664 (53.0)	0.134
Multiparous	1127 (45.5)	539 (44.0)	588 (47.0)	
Miscarriage before child’s birth				
Yes	346 (15.5)	175 (16.1)	179 (15.7)	0.728
No	1886 (84.5)	914 (83.9)	960 (84.3)	
**During Pregnancy Factors**				
Maternal Alcohol during pregnancy				
Yes	43 (1.9)	26 (2.4)	17 (1.5)	0.125
No	2185 (98.1)	1063 (97.6)	1122 (98.5)	
Maternal smoking during pregnancy				
Smoking	354 (15.9)	175 (16.1)	179 (15.7)	0.819
No Smoking	1874 (84.1)	914 (83.9)	960 (84.3)	
High blood pressure during pregnancy	*n* (%)	*n* (%)	*n* (%)	
Yes	69 (3.1)	34 (3.2)	35 (3.1)	0.921
No	2090 (94.6)	1021 (94.7)	1069 (94.5)	
Don’t Know	50 (2.3)	23 (2.1)	27 (2.4)	
GDM during pregnancy				
Yes	54 (2.4)	32 (3.0)	22 (1.9)	**0.033**
No	2100 (95.1)	1027 (95.3)	1073 (94.9)	
Don’t Know	55 (2.5)	19 (1.8)	**36 (3.2)**	
**During Childhood Factors**				
Type of Delivery				
Vaginal	1596 (71.6)	784 (72.0)	812 (71.3)	0.192
Caesarean	632 (28.4)	305 (28.0)	327 (28.7)	
Infant Feeding Practices				
Exclusive Breast feeding	180 (8.1)	90 (8.3)	90 (7.9)	0.695
Exclusive Formula	424 (19.2)	213 (18.6)	211 (18.9)	
Mixed Feeding	1610 (72.7)	775 (71.9)	835 (73.5)	
Solid Food Initiation				
≤4 months	383 (17.3)	185 (17.1)	198 (17.4)	0.935
5–6 months	1476 (66.6)	723 (67.0)	753 (66.3)	
>6 months	356 (16.1)	171 (15.8)	185 (16.3)	
Maternal alcohol during breast feeding				
Yes	22 (1.0%)	15 (1.4)	7 (0.6)	0.069
No	2184 (99.0)	1064 (98.6)	1120 (99.4)	
Maternal smoking during breast feeding				
Yes	21 (1.0)	14 (1.3)	7 (0.6)	0.102
No	2185 (99.0)	1065 (98.7)	1120 (99.4)	
Maternal smoking at their child’s pre-adolescence				
Yes	843 (39.2)	405 (38.8)	438 (39.5)	0.739
No	1308 (60.8)	638 (61.2)	670 (60.5)	
Parents smoking at home, at their child’s pre-adolescence				
Yes	879 (45.1)	422 (44.4)	457 (45.8)	0.517
No	1060 (54.9)	529 (55.6)	540 (54.2)	

Abbreviations: %, percentage; ±, plus/minus; SD, Standard Deviation; AGA, Average for gestational age; SGA, Small for gestational age; LGA, Large for gestational age; GDM, Gestational diabetes mellitus. ^a^ Subsample with selected cases, filtered for energy intake (excluding those with energy intakes of <800 kilocalories (kcal)/day and >3000 kcal/day). ^b^ All *p*-values were derived from chi-square tests and indicate the statistical significance of the differences between boys and girls. Results in bold indicate statistical significance (*p* < 0.05).

**Table 4:** Adjusted ^a^ odds ratios for the association of maternal weight status and child obesity status, stratified by sex.

Maternal Weight Status	Total Sample (OR [95%CI]) (*n* = 2092)	Boys (OR [95%CI]) (*n* = 1020)	Girls (OR [95%CI]) (*n* = 1072)
Maternal Pre-pregnancy BMI			
Healthy Weight	Reference	Reference	Reference
Underweight	0.45 [0.21, 1.00]	0.50 [0.19, 1.30]	0.33 [0.08, 1.44]
Overweight	**1.90 [1.34, 2.70]**	**1.65 [1.00, 2.72]**	**2.43 [1.45, 4.08]**
Obese	**4.16 [2.47, 7.02]**	**3.55 [1.68, 7.51]**	**5.60 [2.60, 12.08]**
Gestational Weight Gain			
Within IOM	Reference	Reference	Reference
Below IOM	0.77 [5.34, 1.10]	0.73 [0.45, 1.19]	0.81 [0.47, 1.40]
Above IOM	**1.50 [1.08, 2.08]**	**1.66 [1.07, 3.56]**	1.45 [0.87, 2.42]
Mothers BMI at their child’s pre-adolescence			
Healthy weight	Reference	Reference	Reference
Under weight	0.24 [0.03, 1.78]	0.36 [0.47, 2.72]	0.00
Overweight	**1.99 [1.45, 2.74]**	**1.87 [1.21, 2.87]**	**2.43 [1.48, 3.98]**
Obese	**3.34 [2.29, 4.87]**	**3.14 [1.89, 5.23]**	**4.18 [2.31, 7.56]**

Abbreviations: OR, Odds Ratio; 95%CI, 95% Confidence Interval; IOM, Institute of Medicine (Based on recommendations by the IOM (2009) report. ^a^ The model was adjusted for infant’s weight at birth (grams), growth velocity, infant feeding history, gestational age, average dietary energy intake (Kcal/day), daily moderate-to-vigorous physical activity (minutes), mother’s age, mother’s education, mother’s age at birth, father’s education, father’s age, and fathers BMI. Subsample with selected cases, filtered for energy intake (excluding those with energy intakes of <800 kilocalories (kcal)/day and >3000 kcal/day). Results in bold indicate statistically significant odds ratios (*p* < 0.05).

## Data Availability

The datasets generated during and/or analysed during the current study are available from the corresponding author on reasonable request.

## Supplementary Materials

The following supporting information can be downloaded at: https://www.mdpi.com/article/10.3390/nu15214602/s1, Figure S1: Maternal weight status across the time-points; pre-pregnancy, gestational weight gain, and at their child’s pre-adolescence; Figure S2: Prevalence of childhood obesity, across recommendation-based maternal weight trajectory groups, stratified by sex; Table S1: Behavioural descriptive characteristics of study participants; Table S2: Crude odds ratios for the association of maternal weight status and child obesity status, stratified by sex; Table S3: Crude odds ratios for the association of recommendation-based maternal weight trajectory groups and child obesity status, stratified by sex; Table S4: Adjusted ^a^ odds ratios for the association of recommendation-based maternal weight trajectory groups and child obesity status, stratified by sex.
