# Supplementary material for: The Association of Maternal Weight Status throughout the Life-Course with the Development of Childhood Obesity: A Secondary Analysis of the Healthy Growth Study Data
Source: Nutrients. 2023 Oct 29;15(21):4602. doi: 10.3390/nu15214602 (PMC10649313; doi:10.3390/nu15214602)
Supplement: Supplementary file 1 [file nutrients-15-04602-s001.zip › nutrients-2669296-supplementary.pdf]

Supplementary Figure S1 Maternal weight status across the time-points; pre-pregnancy, gestational weight gain, and at their child's pre-adolescence.

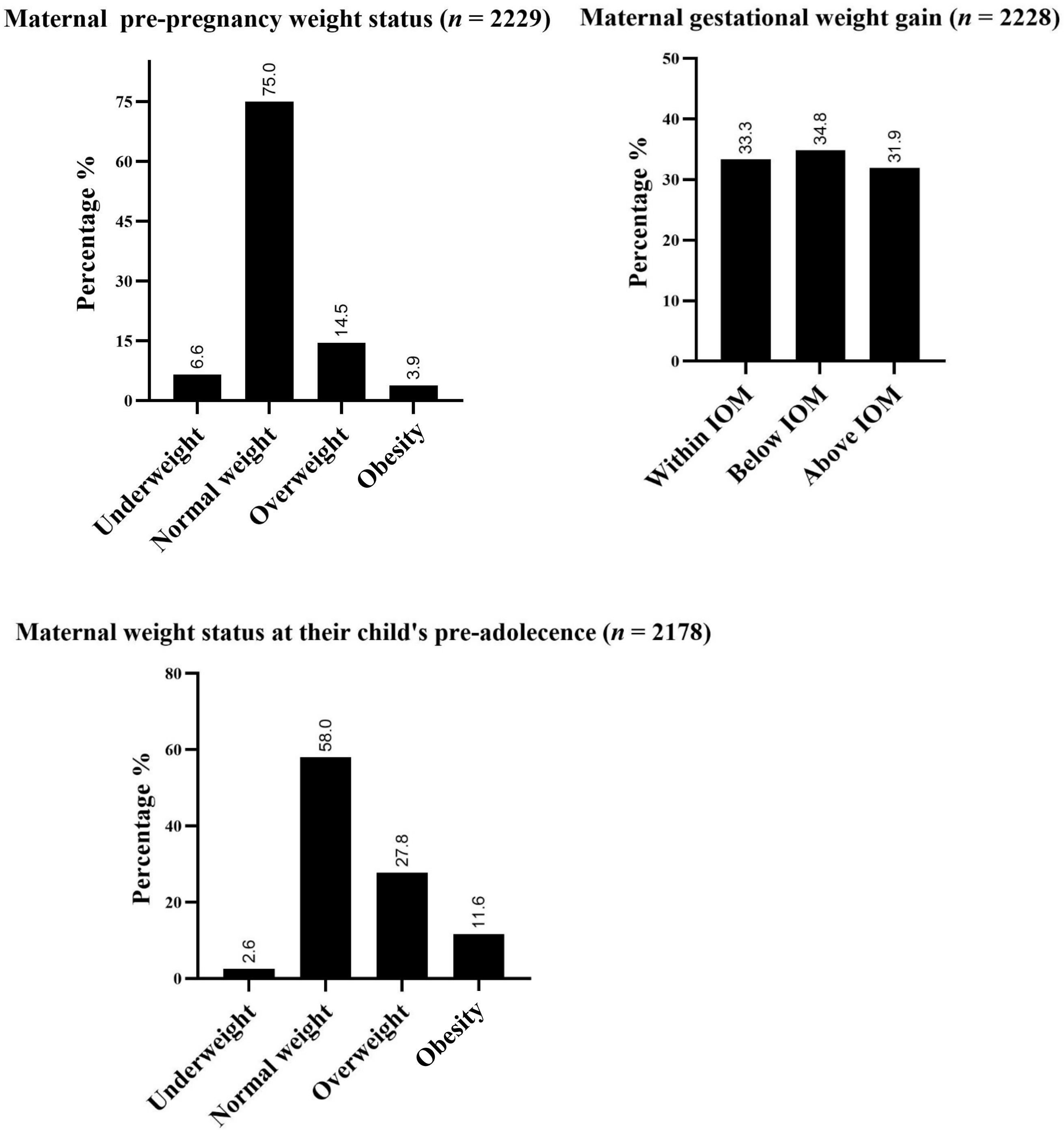

Abbreviations: n, sample size; IOM, Institute of Medicine. *Based on recommendations by the IOM [46].

Supplementary Figure S2 Prevalence of childhood obesity, across recommendation-based maternal weight trajectory groups, stratified by sex.

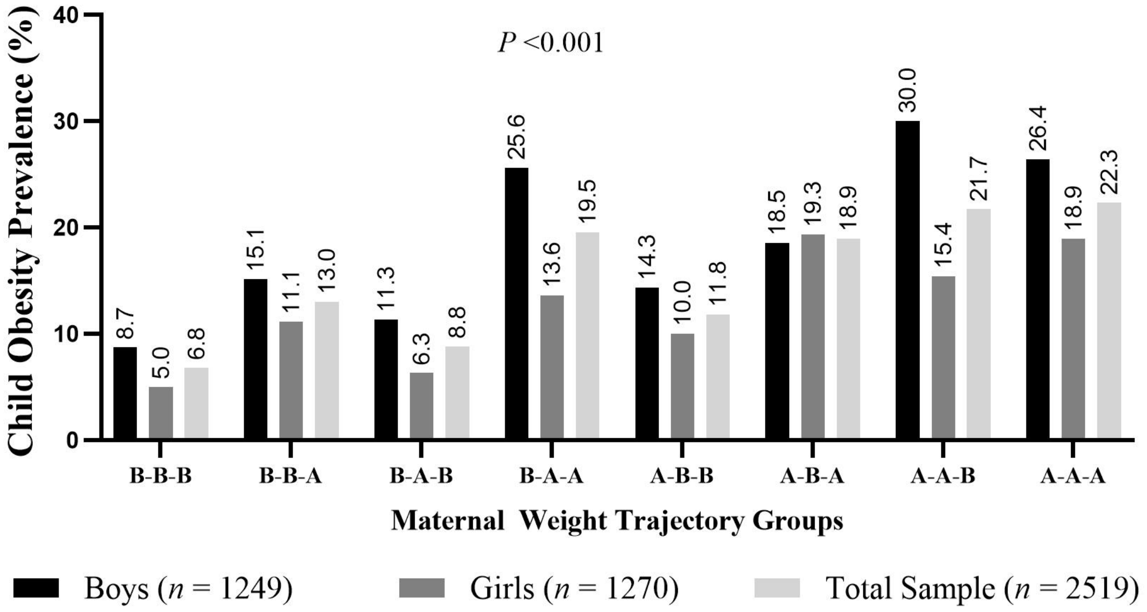

Abbreviations: %, percentage; *P, p* value (determined with a chi-square test); B, Below (includes maternal weight status as normal or below the WHO classification of adults according to BMI [45], or Institute of Medicine (IOM) recommendations [46]); A, Above (includes maternal weight status above the WHO (2000) classification of adults according to BMI thresholds (overweight/obesity) or IOM recommendations [46]); n, sample size.

Maternal weight trajectories groups have been combined based on recommended cut-off points for weight status at the following time-points pre-pregnancy and during pregnancy (gestational weight gain [based on the IOM recommendation [46]]), and, at their child's pre-adolescence, combined into 8 maternal weight trajectory groups.

Supplementary Table S1 Behavioural descriptive characteristics of study participants

	Total Sample (*n* = 2519[a])	Boys (*n* = 1249)	Girls (*n* = 1270)	*P*-value
**Dietary Intake**	Mean ± SD	Mean ± SD	Mean ± SD	
Total energy (kcal/day)	1750.3 ± 450.9	**1829.0 ± 459.6**	**1672.8 ± 428.6**	**<0.001***
% of energy derived from total fat	40.9 ± 7.17	40.6 ± 7.10	41.2 ± 7.24	0.073*
% of energy derived from total SFA intake	15.0 ± 3.51	14.9 ± 3.4	15.1 ± 3.61	0.470*
% of energy derived from total MUFA intake	18.5 ± 5.02	18.4 ± 5.02	15.1 ± 5.02	0.556*
% of energy derived from total PUFA intake	5.17 ± 3.85	**4.90 ± 3.01**	**5.43 ± 4.51**	**<0.001***
% of energy derived from total carbohydrates intake	45.6 ± 8.34	45.7 ± 8.19	45.5 ± 8.50	0.480*
% of energy derived from total protein intake	15.7 ± 3.43	**15.8 ± 3.3**	**15.5 ± 3.58**	**0.002***
**Physical Activity**	Mean ± SD	Mean ± SD	Mean ± SD	
Daily organised moderate-to-vigorous physical activity (min/day)	24.0 ± 38.0	**28.4 ± 45.6**	**19.6 ± 28.6**	**<0.001ᵻ**
Daily moderate-to-vigorous physical activity (min/day)	68.0 ± 63.1	**75.2 ± 65.7**	**58.2 ± 58.0**	**<0.001ᵻ**
Daily steps	13,189.1 ± 5143.0	**14,631.0 ± 5535.3**	**11,782.1 ± 4286.4**	**<0.001ᵻ**
Screen time (hours/day)	3.02 ± 1.77	3.02 ± 1.77	3.02 ± 1.77	0.951ᵻ

Abbreviations: %, percentage; ±, plus/minus; SD, Standard Deviation; SFA, Saturated fat; MUFA, monounsaturated fat; PUFA, Polyunsaturated fatty acids.

[a]Subsample with selected cases, filtered for energy intake >800 <3000 kilocalories.

*All p-values related to gender differences of dietary intake were derived from Independent Sample T-Test using the logarithm of the examined outcome measures.

ᵻAll p-values related to gender differences of physical activity were derived from the non-parametric Mann-Whitney U Test.

Results in bold indicate statistical significance ($p < 0.05$).

Supplementary Table S2 Crude odds ratios for the association of maternal weight status and child obesity status, stratified by sex

Maternal Weight Status	Total Sample* (OR [95%CI])	Boys* (OR [95%CI])	Girls* (OR [95%CI])
Maternal Pre-pregnancy BMI	(n = 2213)	(n = 1082)	(n = 1131)
Normal Weight	Reference	Reference	Reference
Underweight	0.45 [0.21, 0.99]	0.50 [0.199, 1.28]	0.35 [0.09, 1.48]
Overweight	**1.91 [1.37, 2.66]**	**1.71 [1.09, 2.70]**	**2.29 [1.41, 3.71]**
Obesity	**4.20 [2.59, 6.81]**	**3.61 [1.82, 7.11]**	**5.18 [2.58, 10.41]**
Gestational Weight Gain	(n = 2213)	(n = 1082)	(n = 1131)
Within IOM	Reference	Reference	Reference
Below IOM	0.78 [0.55, 1.09]	0.70 [0.45, 1.11]	0.91 [0.54, 1.53]
Above IOM	**1.47 [1.08, 2.00]**	**1.52 [1.01, 2.27]**	1.46 [0.89, 2.37]
Mothers BMI at their child's pre-adolescence	(n = 2162)	(n = 1052)	(n = 1110)
Normal weight	Reference	Reference	Reference
Under weight	0.22 [0.03, 1.61]	0.33 [0.04, 2.49]	0.00
Overweight	**2.06 [1.52, 2.79]**	**1.89 [1.27, 2.83]**	**2.37 [1.48. 3.79]**
Obesity	**3.61 [2.53, 5.16]**	**3.34 [2.06, 5.42]**	**4.18 [2.44, 7.15]**

Abbreviations: OR, Odds Ratio; 95%CI, 95% Confidence Interval; IOM, Institute of Medicine (Based on recommendations by the IOM [46].
*Subsample with selected cases, filtered for energy intake >800 <3000 kilocalories
Results in bold indicate statistically significant odds ratios ($p < 0.05$).

Supplementary Table S3 Crude odds ratios for the association of recommendation-based maternal weight trajectory groups and child obesity

**Maternal Weight Trajectory Group**‡	**Total Sample*** (OR [95%CI]) (n = 2162)	**Boys*** (OR [95%CI]) (n = 1052)	**Girls*** (OR [95%CI]) (n = 1110)
Below-below-below	Reference	Reference	Reference
Below-below-above	**2.04 [1.36, 3.07]**	**1.86 [1.08, 3.19]**	**2.38 [1.30, 4.47]**
Below-above-below	1.31 [0.82, 2.08]	1.33 [0.74, 2.38]	1.27 [0.59, 2.72]
Below-above-above	**3.30 [2.07, 5.26]**	**3.61 [1.98, 6.58]**	**2.99 [1.40, 6.38]**
Above-below-below	1.82 [0.41, 8.12]	1.74 [0.21, 14.84]	2.12 [0.26, 17.39]
Above-below-above	**3.18 [2.01, 5.04]**	**2.38 [1.25, 4.54]**	**4.56 [2.33, 8.91]**
Above-above-below	3.79 [1.36, 10.53]	**4.48 [1.12, 18.00]**	3.46 [0.73, 16.50]
Above-above-above	**3.91 [2.56, 5.96]**	**3.76 [2.12, 6.68]**	**4.43 [2.34, 8.37]**

Abbreviations: OR, Odds Ratio; 95%CI, 95% Confidence Interval; B, Below (includes maternal weight status as normal or below the WHO classification of adults according to BMI [45], or Institute of Medicine (IOM) recommendations [46]); A, Above (includes maternal weight status above the WHO classification of adults according to BMI thresholds (overweight/obesity) [45] or IOM recommendations [46]).
‡Maternal weight trajectories groups have been combined based on recommended cut-off points for weight status at the following time-points pre-pregnancy and during pregnancy (gestational weight gain [based on the IOM recommendation [46]]), and, at their child's pre-adolescence, combined into 8 maternal weight trajectory groups.
*Subsample with selected cases, filtered for energy intake >800 <3000 kilocalories.
Results in bold indicate statistically significant odds ratios ($p < 0.05$).

Supplementary Table S4 Adjusted[a] odds ratios for the association of recommendation-based maternal weight trajectory groups and child obesity status,

Maternal Weight Trajectory Group‡	Total Sample (OR [95%CI]) (n = 2039*)	Boys (OR [95%CI]) (n = 993)	Girls (OR [95%CI]) (n = 1046)
Below-below-below	Reference	Reference	Reference
Below-below-above	**1.86 [1.21, 2.84]**	1.73 [0.98, 3.06]	**2.25 [1.16, 4.39]**
Below-above-below	1.22 [0.75, 1.96]	1.29 [0.70, 2.38]	1.31 [0.60, 2.87]
Below-above-above	**2.90 [1.78, 4.74]**	**3.16 [1.67, 5.99]**	**2.77 [1.23, 6.21]**
Above-below-below	1.61 [0.34, 7.71]	1.07 [0.11, 10.43]	1.96 [0.209, 18.419]
Above-below-above	**2.74 [1.65, 4.54]**	1.88 [0.91, 3.90]	**4.37 [2.09, 9.12]**
Above-above-below	**3.24 [1.10, 9.55]**	3.73 [0.85, 16.31]	3.66 [0.69, 19.39]
Above-above-above	**3.07 [1.95, 4.85]**	**2.43 [1.28, 4.63]**	**4.62 [2.34, 9.12]**

Abbreviations: OR, Odds Ratio; 95% CI, 95% Confidence Interval; B, Below (includes maternal weight status as normal or below the WHO classification of adults according to BMI [45], or Institute of Medicine (IOM) recommendations [46]); A, Above (includes maternal weight status above the WHO classification of adults according to BMI thresholds (overweight/obesity) [45] or IOM recommendations [46]).

[a]The model was adjusted for infant's weight at birth (grams), growth velocity, infant feeding history, gestational age, average dietary energy intake (Kcal/day), daily moderate-to-vigorous physical activity (minutes), mother's age, mother's education, mother's age at birth, father's education, father's age, and fathers BMI.

‡Maternal weight trajectories groups have been combined based on recommended cut-off points for weight status at the following time-points pre-pregnancy and during pregnancy (gestational weight gain [based on the IOM recommendation [46]]), and, at their child's pre-adolescence, combined into 8 maternal weight trajectory groups.

*Subsample with selected cases, filtered for energy intake >800 <3000 kilocalories.

Results in bold indicate statistically significant odds ratios ($p < 0.05$).
